# Seismic air gun exposure during early-stage embryonic development does not negatively affect spiny lobster *Jasus edwardsii* larvae (Decapoda:Palinuridae)

**DOI:** 10.1038/srep22723

**Published:** 2016-03-07

**Authors:** Ryan D. Day, Robert D. McCauley, Quinn P. Fitzgibbon, Jayson M. Semmens

**Affiliations:** 1 Fisheries and Aquaculture Centre, Institute for Marine and Antarctic Studies, University of Tasmania, Hobart, Tasmania, Australia; 2 Centre for Marine Science and Technology, Curtin University, Perth, Western Australia, Australia

## Abstract

Marine seismic surveys are used to explore for sub-seafloor oil and gas deposits. These surveys are conducted using air guns, which release compressed air to create intense sound impulses, which are repeated around every 8–12 seconds and can travel large distances in the water column. Considering the ubiquitous worldwide distribution of seismic surveys, the potential impact of exposure on marine invertebrates is poorly understood. In this study, egg-bearing female spiny lobsters (*Jasus edwardsii*) were exposed to signals from three air gun configurations, all of which exceeded sound exposure levels (SEL) of 185 dB re 1 μPa^2^·s. Lobsters were maintained until their eggs hatched and the larvae were then counted for fecundity, assessed for abnormal morphology using measurements of larval length and width, tested for larval competency using an established activity test and measured for energy content. Overall there were no differences in the quantity or quality of hatched larvae, indicating that the condition and development of spiny lobster embryos were not adversely affected by air gun exposure. These results suggest that embryonic spiny lobster are resilient to air gun signals and highlight the caution necessary in extrapolating results from the laboratory to real world scenarios or across life history stages.

Anthropogenic noise has shown the potential to negatively affect animals from arthropods to mammals through the disruption of fundamental biological processes such as metabolism, immune function, reproduction and development^1^. The impacts of anthropogenic noise in aquatic environments are of particular concern^2^,^3^,^4^ as sound travels farther, faster and more efficiently (i.e. lower attenuation of intensity) in water than through air^5^, resulting in a greater area of potential impact.

A major source of anthropogenic noise in the marine environment is the use of seismic air guns for oil and gas exploration. Air guns represent a technological advancement offering an apparent improvement in animal welfare over the effects of previous methods, such as the use of explosives, which show a distance dependent spectrum of impact ranging from mortality at close range to organ damage, sensory disruption and behavioural alterations at increasing distances from the source^6^. However, concerns over the effects of air gun signals on wildlife remain, as marine mammals^7^ and fishes^8^,^9^,^10^,^11^ have been shown to demonstrate altered behaviour and physiology following exposure. Economic concerns have also been raised over reduced abundance and catch rates reported during and immediately following seismic surveys for a variety of fisheries species, e.g. blue whiting (*Merlangus merlangus*)^12^, rockfish (*Sebastes* spp.)^13^, cod (*Gadus morhua*) and haddock (*Melanogrammus aeglefinus*)^14^, herring (*Clupea* spp.)^15^, American lobster (*Homarus americanus*)^16^ and snow crab (*Chionoecetes opilio*)^17^.

Despite their ecological and socioeconomic importance, comparatively little is known about the impact of seismic surveys on marine invertebrates. A recent gap analysis by Hawkins *et al.*^18^ highlighted a range of issues to be addressed before conclusions can be drawn by researchers, industries and regulatory bodies. These issues range from improving the current understanding of the sources of aquatic noise and the methods and metrics used to quantify exposure, to the characterisation of sound propagation through the water, and the ability of marine invertebrates to produce and even sense sound. It is not surprising, given these substantial gaps in knowledge, that industry groups representing commercially important invertebrates such as spiny lobsters^19^ and scallops have cited concern over seismic surveys resulting in mass deaths^19^,^20^, with one such incident blamed by industry groups for the loss of AU$70 million worth of scallops.

An understanding of the effects of anthropogenic noise in general on the early life history stages of marine invertebrates is still developing, whereas specific knowledge of the effects of air gun exposure is nearly non-existent. Laboratory based exposure to aquatic noise approximating (but notably, not emulating) a seismic survey had a catastrophic effect on scallop (*Pecten novazelandiae*) larvae characterised by abnormal morphological development^21^. However, the applicability of these laboratory assessments to *in situ* seismic surveying is unclear, as acoustic studies conducted in laboratory tanks have been discouraged for half a century^22^,^23^,^24^ owing to an inability to understand what stimulus animals in the tank are actually exposed to, a result of the physics of generating signals and long wavelength sound in small, reflective tanks. Two field based studies conducted on early-stage crustaceans have shown that exposure to seismic air guns had no effect on Dungeness crab (*Cancer magister*) zoaea^25^ and significantly increased egg mortality and delayed development in snow crab (*C. opilio*) eggs^17^. Clearly, given the almost complete lack of research; the contradictory results of what little research has been conducted; the change in sensory capability for a species during development; and the considerable diversity within, and substantial differences between, the molluscan phylum and the crustacean subphylum, drawing any sort of conclusion on the developmental, physiological, ecological impacts of exposure to seismic air gun signals on marine invertebrates is not possible. Without a better understanding of the effects and impacts of exposure to seismic air gun signals, evidence based management and regulation decisions cannot be made and any claims of financial loss following surveys are impossible to substantiate or refute.

In light of the substantial gaps in knowledge, the confounding methods employed by previous studies and the subsequent conflicting results, the present study investigates the impacts of seismic air gun exposure on the spiny lobster (*Jasus edwardsii*). The spiny lobster from the family Palinuridae is a useful model for marine invertebrates, as it is the most valuable single species capture fishery in Australia and spiny lobsters are amongst the most valuable fishery species worldwide^26^, with an annual catch of over 81,000 tonnes in 90 countries worth an estimated US$775 million^27^. If seismic air gun exposure causes maternal stress^28^, egg mortality, delayed development or severe morphological abnormality^17^,^21^, this ecologically and financially important decapod crustacean family could be devastated by compromised reproductive output and reduced recruitment.

Here, we show that exposure to air gun signals during the embryonic stage does not detrimentally effect spiny lobster development, as the resultant exposed larvae did not differ from control larvae, either in terms of quantity or quality. Furthermore, we present seismic data demonstrating that our approach using three air gun configurations in a field-based, natural lobster habitat resulted in exposure equivalent to real-world seismic surveys. Finally, we discuss these results relative to the few previous studies of seismic exposure in early life history stages of other marine invertebrates, with a focus on methodical differences and the implications for extrapolating experimental results into real world settings.

## Results

### Seismic exposure

**Figure 1:**
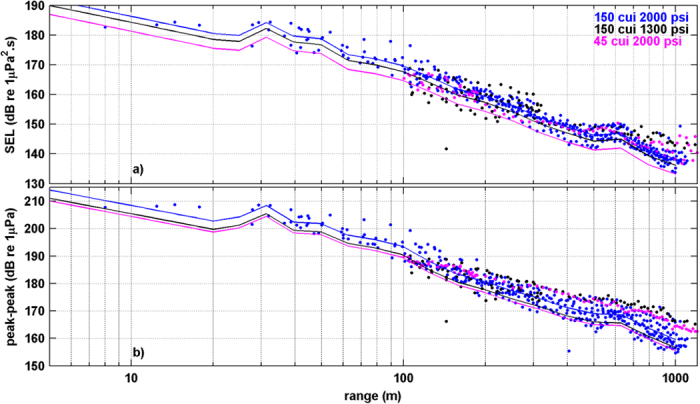
Quantification of sound exposure by range between the air gun vessel and the lobster pots in the 45 in^3^, 150 in^3^ low pressure and 150 in^3^ high pressure air gun exposure experiments. Sound level is expressed in received sound exposure level (**a**) and received peak-peak level (**b**) in the three trials with range expressed logarithmically.

Air gun runs were made starting at 1–1.5 km from the line of pots with the source run towards and over the pots, with total air gun exposures of 24.3, 17.2 and 23.3 minutes, for 126, 112 and 110 shots for the 45 in^3^, 150 in^3^ low pressure and 150 in^3^ high pressure experiments. Control runs emulated the exposure runs with the source deployed and pressurised but not operated. Estimates of sound exposure (SEL) and peak-to-peak level for the pressure component of each air gun signal were made at all lobster pots using empirical measures made in the field at the seabed (where the lobsters were held), adjusted for air gun source levels (see Supplementary Information). Estimates of received signal levels (peak-to-peak and sound exposure level) were made for each pot then statistics of the maximum and ‘average’ exposure made using all pots per experiment. The air gun source levels (at 1 m reference distance) were: 223, 224 and 227 dB re 1 μPa for peak-to-peak and 200, 203, 205 dB re 1 μPa^2^·s for SEL source levels in the 45 in^3^, 150 in^3^ low pressure and 150 in^3^ high pressure experiments, respectively (Fig. 1). The 150 in^3^ low pressure experiment functioned as a moderate exposure level relative to the lower intensity 45 in^3^ and the higher intensity 150 in^3^ high pressure experiments, thus increasing the spread of exposures.

Maximum exposure received at any pot was dependent on proximity to the air gun which was random amongst experiments, but the cumulative sound loading, or total dose of sound received per experiment, was lowest for the 45 in^3^ experiment, intermediate for the 150 in^3^ low pressure experiment and highest for the 150 in^3^ high pressure experiment as given by the number of signals which exceeded set thresholds and the maximum or median cumulative SEL_cum_ (defined as the sum of the linear value of each shot’s sound exposure intensity, converted back to a dB value, see Table 1 for values). The maximum and median cumulative sound exposure level estimated in the three experimental regimes were 192 and 191 for the 45 in^3^ experiment, 193 and 192 for the 150 in^3^ low pressure experiment and 199 and 197 dB re 1 μPa^2^·s for the 150 in^3^ high pressure experiment, while the maximum number of shots amongst pots exceeding 180 dB re 1 μPa^2^·s differed substantially, at 3, 7 and 25 for the 45 in^3^, 150 in^3^ low pressure and 150 in^3^ high pressure exposures, respectively (Table 1).

### Hatching and fecundity

There were no mortalities of the adult berried female lobsters in either control or exposed treatments for any of the three experiments. Similarly, all females had successful hatches with no incidence of loss or removal of the egg bundle. Lobsters in both treatments over all three experiments hatched over the course of a 5–6 day period, with a peak in the number of larvae hatched around days 3–4.

**Figure 2:**
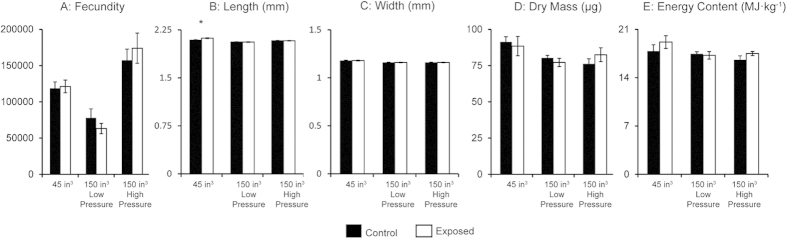
Comparisons of measurements of newly hatched spiny lobster (*Jasus edwardsii*) larvae from 45 in^3^, 150 in^3^ low pressure and 150 in^3^ high pressure air gun exposure experiments. Mean larval (**A**) fecundity, (**B**) length, (**C**) width, (**D**) dry mass and (**E**) energy content with error bars indicating SEM. Larval length was significantly different between control and exposed treatments for the 45 in^3^ experiment as determined using a nested ANOVA and is indicated with an asterisk. Statistically significant differences between experiments are not shown.

Comparison of the number of larvae hatched (Fig. 2A) between all treatments using ANCOVA with carapace length (CL) as the covariate showed that the mean number of hatched larvae differed significantly (F(5,46) = 4.437, P < 0.003) with CL significantly related to fecundity (F(1,46) = 14.123, P < 0.001). However, differences in fecundity were limited to comparisons between experiments, with no differences between control and exposed treatments within an experiment (45 in^3^ P = 1.00, 150 in^3^ low pressure P = 0.753, 150 in^3^ high pressure P = 0.870).

### Morphology

Observation of larval morphology revealed no abnormalities in any of the hatches. Comparisons of larval body length (Fig. 2B) using nested ANOVA showed significant differences (F(5,47) = 22.52, P < 0.001) between treatments. Tukey HSD post hoc analysis showed a significant difference (P < 0.001) between control and exposed larvae in the 45 in^3^ experiment, as exposed larvae were approximately 1.5% longer than control. When compared between experiments, control larvae from the 45 in^3^ experiment were significantly longer than both control (P < 0.001) and exposed (P < 0.001) treatments from the 150 in^3^ low pressure experiment and exposed larvae from the 45 in^3^ experiment were significantly longer than larvae from any of the other treatments (P < 0.001 for all comparisons). There were no differences in larval length between control and exposed treatments in the 150 in^3^ experiments.

Larval width (Fig. 2C) also showed a significant difference (F(5,47) = 15.192, P < 0.001) when compared using nested ANOVA with Tukey HSD post hoc comparisons. In this case, no differences were found between control and exposed treatments within any of the three experiments. Comparisons between the three experiments showed that larvae from the control treatment of the 45 in^3^ experiment had a significantly greater width than larvae from both treatments of the 150 in^3^ low pressure experiment (control P < 0.001, exposed P < 0.001) and from both treatments of the 150 in^3^ high pressure experiment (control P < 0.005, exposed P < 0.002). Larvae from the exposed treatment of the 45 in^3^ experiment were significantly wider than larvae from both treatments of the 150 in^3^ low pressure experiment (control P < 0.001, exposed P < 0.001) and from both treatments of the 150 in^3^ high pressure experiment (control P < 0.001, exposed P < 0.001).

Length-to-weight and width-to-weight ratios were compared between treatments for all three experiments; however, as there were no differences apparent, these data are not shown.

### Dry Mass and Energy

Contrary to the results of larval length and width comparisons, no significant differences were found within or between the dry masses (Fig. 2D) of any of the treatments (F(5,49) = 1.751, P < 0.15). Similarly, larval energy content (Fig. 2E) did not differ between treatments in any of the exposure levels when compared using ANOVA (F(5,44) = 1.493, P < 0.212).

### Competency

**Figure 3:**
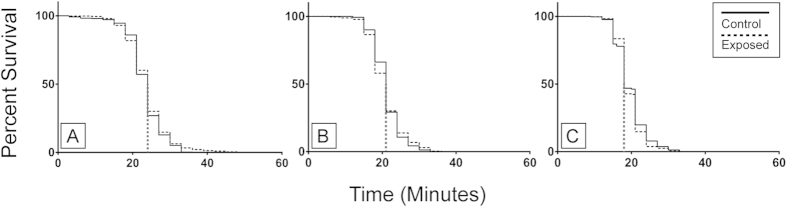
Competency of newly hatched spiny lobster (*Jasus edwardsii*) larvae from 45 in^3^ (**A**), 150 in^3^ low pressure (**B**) and 150 in^3^ high pressure (**C**) air gun exposure experiments. Kaplan-Meier survival analysis of larval activity test indicates the estimated percentage of larvae surviving over the test time period, in minutes, for control and exposed larvae. Median survival time for each experiment, which in all cases is the same for control and exposed treatments within the experiment, is indicated by a vertical grey dashed line. For the 45 in^3^ experiment, each curve represents 600 larvae. For the 150 in^3^ low pressure experiment the control curve represents 400 larvae and the exposed curve represents 600 larvae. For the 150 in^3^ high pressure experiment, the control curve represents 480 larvae and the exposed curve represents 400 larvae. For all three experiments, the lack of any difference in both the median survival time and the slope of the curves representing the two treatments indicates that there was no difference in level of larval competency.

No difference was found in larval competency, as measured through an elevated temperature and reduced salinity activity test^29^, between control and exposed larval treatments from the 45 in^3^ experiment (Fig. 3A). Both treatments had a median survival time of 24 min and the hazard ratio, which compares the slope of the survival curves and thus the rate of death, was 1.129 with a 95% confidence interval (95%CI) of 0.9742, 1.308 for control larvae and 0.8860 with a 95%CI of 0.7647, 1.026 for exposed larvae. These hazard ratio results reflect the proportion of deaths occurring at any given point in one treatment relative to the other treatment—i.e. at any given time, the probability of a control larvae death was 1.129 times that of an exposed larvae. Again, there was no difference in the activity test results between control and exposed larvae from the 150 in^3^ low pressure experiment (Fig. 3B). Both treatments had a 21 min mean survival time and a hazard ratio of 1.002 with a 95%CI of 0.8846, 1.139 for control larvae and 0.9978 with a 95%CI of 0.8777, 1.131 for exposed. Similarly, no difference was found in activity results for 150 in^3^ high pressure larvae (Fig. 3C), with median survival of 18 min for both control and exposed larvae and hazard ratio of 0.9397 95%CI 0.7795, 1.017 for control and 1.064 95%CI 0.9829, 1.283 for exposed treatments.

## Discussion

This study investigated the effects of seismic air gun signal exposure on spiny lobster embryonic development, as assessed through the number, morphology, energy content and competency of hatched larvae. The air gun exposure regime gave a spread of comparatively high sound loadings at the received lobster, with estimates of the sum of sound exposure of all received air gun shots yielding median cumulative sound exposure (SEL_cum_) values of 190, 191 and 197 dB re 1 μPa^2^·s amongst replicates for the 45 in^3^, 150 in^3^ low pressure and 150 in^3^ high pressure experiments respectively). Putting this exposure into context is somewhat difficult, as there are few published values for comparison. A 3590 in^3^ commercial array operating in 990 m water with a receiver 250 m off the bottom measured a maximum SEL of approximately 178 dB dB re 1 μPa^2^·s and a SEL_cum_ of 187 dB re 1 μPa^2^·s^30^. Measurements of 3040 in^3^ and 2130 in^3^ arrays operating in 152 m depth recorded maximum SEL values of 178 and 174 dB re 1 μPa^2^·s and SEL_cum_ values of 189 and 188 dB re 1 μPa^2^·s respectively^31^. A 3130 in^3^ array operating in a water depth of 36 m depth recorded SEL values of 172 dB re 1 μPa^2^·s and SEL_cum_ values of 190 dB re 1 μPa^2^·s at 500 m range (RM, unpublished data). Thus, the SEL_cum_ values of 191–197 dB re 1 μPa^2^·s recorded in the present study emulate exposures equivalent to those of a large commercial air gun array passing within a few hundred m and certainly <500 m, of the experimental site.

To assess the biological impact of air gun exposure, three primary concerns were investigated. The first was the loss of eggs either through direct mortality or caused by over-grooming of the egg bundle by the female, which is a known behavioural response to stress^28^. This concern was not supported, as exposure to signals from seismic air guns did not result in any apparent egg bundle loss, nor were there any differences in fecundity between control and exposed lobsters from any of the three exposure levels. The fecundity of the lobsters used in this study was on par with that of previous reports for similar sized *J. edwardsii*^32^,^33^. The only observed differences in fecundity were between experiments, with both control and exposed treatments in the 150 in^3^ low pressure experiment hatching significantly less larvae than in the other two experiments. However, given the lack of difference between control and exposed treatments in this experiment, along with the fact that lobsters for this experiment were collected from the same site as the 45 in^3^ experiment and were approximately the same age (based on carapace length), this low fecundity relative to that of lobsters exposed to a lower SEL in the 2013 experiment and a higher SEL in the 2014 high pressure experiment cannot be attributed to air gun exposure. Based on the consistency in the collection, transportation and animal husbandry methods between experiments and the consideration that the females were berried prior to collection for the experiment, the most parsimonious explanation for this result is natural variation in clutch size.

The second primary concern regarded the quality of the larvae, with *a priori* expectations that exposure may result in reduced larval energy content or larval competency, as assessed using a well-established activity test developed on *J. edwardsii* larvae that correlates activity in a reduced salinity, increased temperature environment with the rate of survival through phyllosoma moulting stages^29^. Again, this concern was not supported, as no difference was found in either larval energy or competency at any of the three levels of exposure.

The third concern, that exposure would result in abnormal larval morphology, cannot be immediately dismissed. Although no apparent morphological abnormalities were observed, exposed larvae from the 45 in^3^ experiment were found to be significantly longer than control larvae.

Larval length in crustaceans shows a substantial degree of natural variability and can be affected by a range of factors^34^,^35^, including biotic influences such as maternal size and maturity^36^,^37^ and abiotic factors such as differences in temperature^29^,^33^,^38^ and photoperiod^38^,^39^. Indeed, the size of larvae in this study fall well within the range for Stage I larval length of *J. edwardsii* reported by Lesser^40^, indicating that the range of natural variation in larvae is much greater that the differences observed between treatments in this study. Furthermore, these morphological differences were not found to translate to any difference in either larval energy content or competency despite the expectation that larger larvae should be more competent than smaller larvae^33^.

Whether or not the observed differences in size are biologically significant, seismic exposure did not result in a decrease in fecundity, either through a reduction in the average number of hatched larvae or as a result of high larval mortality; compromised larvae or morphological abnormalities, thus none of the three concerns over embryonic exposure to seismic air gun signals were supported. These results support the suggestion that early life stage crustaceans may be more resilient to seismic air gun exposure than other marine organisms^25^.

Indeed, the evidence suggesting seismic exposure negatively affects the embryos of marine invertebrates is limited and questions must be raised regarding the methods of these studies. A recent study of New Zealand scallops (*P. novazelandiae*) exposed to recordings of an air gun played using an acoustic projector in a tank found larvae hatched following embryonic sound exposure suffered significantly delayed development and a nearly 50% occurrence of growth abnormality^21^. Based on these results, the authors raised concerns about the impacts of seismic exploration in spawning areas of marine invertebrates. However, the results from acoustic work in tanks cannot be put into real world context, as the long wavelengths produced by real sources such as an air gun cannot be emulated in a small tank. First, real sources cannot be used in tanks, creating a problem in emulating the physics of the source. Second, sound bounces off tank surfaces, resulting in large amounts of constructive or destructive interference at small spatial scales, as well as the creation of a complex and unpredictable relationship between sound pressure and particle motion^41^. Similarly, experiments have been performed in extremely shallow water depths e.g. refs 16,25, which risks overestimation of the level of acoustic energy experimental animals receive as phase cancellation creates a “sound shadow” resultant from sound waves reflecting from the water’s surface^42^,^43^. Finally, methods must be either biologically relevant or experimentally validated if results are to be extrapolated to real world conditions. Seismic exposure was suggested to result in significantly higher rates of mortality and significantly delayed development in snow crab (*C. opilio*) embryos^17^, however, this experiment was performed on eggs stripped from the females and cultured in a laboratory for six weeks prior to exposure and eighteen weeks following exposure. Subsequent work on larvae that had been exposed to air gun signals as embryos but were allowed to hatch normally without being stripped from berried females did not suffer any negative effects^44^. In light of the emerging trend in which the deleterious results observed in laboratory studies are not supported by the results of field based experiments, it is apparent that results from the field are necessary before laboratory studies can be relied upon to supplement our understanding of effects in the field and inform any meaningful conclusions of seismic air gun exposure.

It must be noted that, at the time of exposure in the present study, the spiny lobster eggs were at an early embryonic developmental stage, just after extrusion and prior to eye development, and were thus entirely soft tissue with no large internal density differences. Such large internal density differences could cause localised transfer of high intensity acoustic energy to physical forces within the egg. Later spiny lobster larval developmental stages have developed sensory systems including arrays of pinnate setae along the flagella of the antennae and mechanosensory statocyst organs which they may use for navigation during the critical onshore migration and settlement phase i.e. refs 45,46. As such, the experimental results found here may not necessarily be the same for spiny lobsters exposed later in development (including later stage embryos, larvae and adults) and is an area which requires further research to determine the potential impacts of seismic surveys on lobster populations. Until such information is available, an inability to draw conclusions on the effects of air gun exposure will persist, preventing the development of evidence-based regulation for seismic surveys.

It is clear that the current understanding of the impacts seismic air gun signals may have on early life history stage marine invertebrates is limited. In light of such a limitation, it is necessary to resist misapplying research results and extrapolating laboratory conditions to real world scenarios or across untested life history stages. Although the logistical difficulties and financial imposts of field based experiments present a substantial barrier, results from realistic and representative exposure regimes are necessary to form an accurate understanding of how marine invertebrates are affected by air gun signals. Unlike numerous previous efforts, this study was performed in field settings with an air gun typical of real world surveys. Furthermore, by employing air gun configurations of three different capacities, this study addresses whether the response is dose-dependent, an important factor to scaling the level of exposure to different air gun arrays, operating depths and seabed compositions. Although the results of this study eliminate concern over exposure of lobster embryos early in development, other life stages require investigation before concern over the potential of seismic air gun exposure damaging important invertebrate fisheries can be dismissed entirely.

## Materials and Methods

### Animals

In June 2013, 20 berried, female spiny lobsters (*Jasus edwardsii*) with a mean carapace length (CL) of 95.5 ± 1.3 mm were obtained for the 45 in^3^ air gun (see below) experiment from commercial fishermen from several sites around Shoemaker Point, Tasmania (43° 35′ 38.23″S, 146° 38′ 03.69″E). In July 2014, 17 berried female lobsters with a mean CL of 91.7 ± 1.4 mm were obtained for the 150 in^3^ low pressure air gun experiment (see below) from commercial fishermen from approximately the same sites. For the 150 in^3^ high pressure air gun experiment, 16 berried female lobsters with a mean CL of 105.2 ± 2.1 mm were obtained from the Crayfish Point Scientific Reserve (42° 57′ 10.63″S, 147° 21′ 17.42″E), also in July 2014. Lobsters were randomly allocated into control and exposed treatments (45 in^3^ control *n* = 10, exposed *n* = 10; 150 in^3^ low pressure control *n* = 7, exposed *n* = 10; 150 in^3^ high pressure control *n* = 8, exposed *n* = 8), tagged with an antenna tag and housed in one of four 3400 litre (2 m × 2 m × 0.85 m) holding tanks at the Institute for Marine and Antarctic Studies, Taroona, Tasmania, Australia with each tank receiving ambient temperature seawater filtered in series through a 100 and a 50 μm filter. Lobsters were housed for 5 days prior to transportation to the experimental site. During acclimation and post exposure holding (mean duration: 87 ± 2 days in 45 in^3^ experiment, 79 ± 2 days in 150 in^3^ low pressure experiment, 79 ± 3 days in 150 in^3^ high pressure experiment) they were fed live blue mussels (*Mytilus galloprovincialis*) ad libitum twice weekly.

### Air gun exposure

The study site was over a shallow limestone reef platform with uniform depth of 10–12 m, located north of Betsey Island in Storm Bay, Tasmania (43° 02′ 119″E 147° 28′ 36″S). Lobsters were transported in seawater aerated with O_2_ to maintain 100% saturation and were then placed into lobster pots (n = 20; 760 mm × 760 mm × 440 mm) modified to have a soft mesh bottom to allow for contact with the substrate and an acrylic top panel to prevent lobsters climbing to the top side of the pot during air gun passes. Into each pot, 5 lobsters were placed (1 for the experiments in this study and an additional 4 used for other experiments) and the pots were lowered onto a rocky reef at a depth of 10–12 meters. For the 45 in^3^ air gun and the 150 in^3^ air gun high pressure experiments, the lobsters were left for two days to acclimate after transportation. For the 150 in^3^ low pressure experiment, the acclimation period was extended to 7 days due to technical and weather issues. Prior to the experiment, divers checked the positioning of the pots to ensure they were oriented correctly and in contact with rocky substrate. In both 150 in^3^ experiments, lobsters were lost during the acclimation period due to suspected predation by seals and/or sharks.

The same air gun was used in all three experiments: a Sercel G Gun II with either a 45 in^3^ chamber or a 150 in^3^ chamber and operated at either 2000 psi (45 in^3^ and 150 in^3^ high pressure experiments) or 1300 psi (150 in^3^ low pressure experiment), with the different pressures in the 150 in^3^ experiments to facilitate a greater spread in exposure levels. Additional details of the air gun set-up may be found in the Supplementary Information.

For the three experiments, the air gun vessel began each run from a position 1 km west of the study site with the air gun deployed and then towed at a mean speed of 1.85 ms^−1^ at 5 m depth toward the study site and then along the two parallel lines of lobster pots containing study animals (see Supplementary Information for additional details of air gun runs, lobster pots and noise loggers). For control treatments, the same vessel track was followed with the air gun deployed and fully pressurised, but not fired. For exposed treatments, the air gun was fired every 11.6 s^47^. In all three experiments, the control run was conducted first, after which, 10 lobster pots were randomly selected and recovered to comprise the control treatment. Next, the exposed treatment run was conducted with the seismic vessel following a similar approach and circling over the two parallel lines of lobster pots. At the conclusion of the exposed run, the remaining pots were recovered.

A near field hydrophone was located 0.5 m off the gun ports and all near field air gun signals logged to a digital recorder, using a −20 dB pre-amplifier and −6 dB gain on the recorder and 24 bit, 48 kHz sampling. To monitor the air gun signal exposure received by target animals and the normal ambient noise regime at the site, sea noise loggers were set on the seabed for the full experimental duration, including acclimatisation periods. The configuration and sampling regimes of the noise loggers used are listed in the Supplementary Information. All noise loggers had pressure sensors fitted using High Tek HTI U90 or Massa TR1025C hydrophones.

### Air gun signal analysis and units

All air gun and spatial analysis has been carried out in the Matlab environment using purpose built software. Air gun signals were analysed by: 1) extracting the signals from the sea noise logger files; 2) converting volts to sound pressure (Pa) using the system calibration curve and hydrophone sensitivity in the time domain; 3) characterising the air gun signal for 16 signal parameters as defined in McCauley *et al.*^48^; and 4) aligning the shot received time with the source navigation data to give the source-receiver, slant-range (direct path source to receiver, not horizontal range). A curve was fitted to the measured levels (peak-peak and sound exposure level independently) of the 150 cui high pressure data, using: a) the mean value in logarithmic range bins; and b) of the form


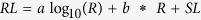


where *RL* is received level, *R* is range, *SL* is the (fixed) source level and *a* & *b* are values derived from the data. The measured curve a) above described the anomalies in the transmission for the site (due to environmental factors) but was less accurate at ranges <20 m where the data was scarce. For peak-peak and SEL the two curves a) and b) agreed over the range 10–20 m so a hybrid curve was used, with ranges <20 m using the curve b) and ranges >20 m using curve a). Each curve was then adjusted for the difference in source level according to the air gun source model to give six sets of curves to predict peak-peak and SEL for the three sources. These curves are shown on Fig. 1.

The range of source to receiver (lobster pot) was then used to estimate received level (peak-peak and SEL) for each shot, at each pot, during each experiment from which the statistics given in Table 1 were derived (noting these were derived using statistics of shots received at individual pots, not using all signals from all pots to give statistics). The cumulative SEL (SEL_cum_ or sum of sound exposure values in linear units of all air gun shots received at a pot, expressed in dB values) were calculated for each pot, with the median and maximum SEL_cum_ values derived using data for the different pots.

Following the control and exposure runs, the lobster pots were recovered and the lobsters were transported back to the facility and returned into the holding tanks and maintained as they were prior to the experiment until hatching which occurred a mean 87 ± 2, 79 ± 2 and 79 ± 3 days post-exposure in the 45 in^3^, 150 in^3^ low pressure and 150 in^3^ high pressure experiments, respectively.

### Hatching

Just prior to larval hatching, as determined by eye index aging^33^, lobsters were moved from communal housing to 20 L isolation tanks with 300 mm × 150 mm panels of 100 μm mesh^29^ to allow for collection of larvae from each individual. Each isolation tank received flow of filtered seawater at ambient temperatures. Isolation tanks were checked daily for hatches, which were drained into a graduated 20 L vessel for subsequent analysis.

### Fecundity

Counts of hatched larvae were performed for each individual on every day hatched larvae were present. To count larvae, the volumetric estimate described by Smith and Ritar^28^ was used. Briefly, larvae were placed into a known volume of water (10, 15 or 20 L, depending on visual estimation of larval density). Larvae were suspended via thorough mixing of the water to ensure an even distribution. Water samples (*n* = 5) of volumes inversely proportional to larval density (50, 125 or 250 ml) were taken and the larvae contained in each sample counted while the sample was decanted into a beaker. The mean number of larvae from the 5 samples was averaged to provide a hatch count.

### Morphometrics

On the first day of an observed hatch, around 40 larvae from each individual were collected and placed between two petri dishes which were then gently pressed to displace excess water and keep larvae prostrate and planar, allowing for measurements to be made using a projection microscope at 20× magnification. From each sample 20 larvae were measured for length and width to the nearest mm (±0.5 mm). Any larvae that were not lying prostrate were not measured, as a prostrate posture was necessary for accuracy. Any naupliosoma that had not yet metamorphosed into larvae were not measured, as the naupliosoma stage is a transient, pre-larval stage that lasts for 30 minutes or less^49^ and has a curled or folded posture that prevents accurate measurement. During the measurement process, larvae were observed for any apparent morphological abnormality. Larval morphological measurement confirmed that all observations were conducted during the first instar phyllosoma stage as described by Lesser^40^.

### Calorimetry

On the third day of hatching, 120 larvae from each hatch were counted, collected into 5 ml sample tubes and snap frozen using liquid nitrogen. Tubes were stored in either liquid nitrogen or a −80° freezer until they were freeze dried. Following the freeze drying process, tubes were sealed and stored in a −80° freezer. To measure the caloric content of each sample, the freeze dried larvae were weighed to the nearest 0.01 mg and measured for energy using a microcalorimeter according to the manufacturer instructions.

### Activity

The competency of the hatched larvae was tested on the second day of each hatch using the activity test described by Smith *et al.*^29^. Briefly, 20 larvae from each hatch were placed into 200 ml plastic sample jars containing 10% seawater held at 21 °C using a heated water bath. Larvae were observed at 3 min intervals and the number of larvae prostrated on the bottom was recorded until no larvae remained active. The number of prostrate larvae within each time interval was averaged for the 3 replicates and used for Kaplan-Maier survival analysis.

### Statistics

Length and width data were tested for normality using the Wilks-Shapiro test and for equality of variances using Bartlett’s test and residual versus fit plots. Length data for all three experiments failed the assumption of normality so empirical Box-Cox transformations were applied^50^. Values of λ for the transformations of length on 45 in^3^, 150 in^3^ low pressure and 150 in^3^ high pressure air gun experiments were 1.5, 1.8 and 1.6, respectively. Width data for all three experiments passed both normality and equality of variance tests, so were not transformed. Data were then analysed using a nested ANOVA with clutch (larvae hatched from the same individual) nested within treatment (control or exposed).

The number of hatched larvae, dry mass and energy comparisons were tested for normality and equality of variance using the Wilks-Shapiro test and Bartlett’s test, respectively. All data sets were normal with equal variances and were analysed first with ANCOVA with carapace length as a covariate. Carapace length was a significant factor only for the count data, so these results are reported, and ANOVA was used to compare dry mass and energy.

All above statistical analyses were performed using R 3.1.3 (The R Foundation for Statistical Computing) calculated at the 5% significance level (α = 0.05)

Larval competency as measured using an elevated temperature and decreased salinity activity test was compared using survival analysis with a Kaplan-Maier estimation and logrank test for trend in GraphPad Prism 6 (GraphPad Software, Inc).

## Additional Information

**How to cite this article**: Day, R. D. *et al.* Seismic air gun exposure during early-stage embryonic development does not negatively affect spiny lobster *Jasus edwardsii* larvae (Decapoda:Palinuridae). *Sci. Rep.* **6**, 22723; doi: 10.1038/srep22723 (2016).

## Supplementary Material

Supplementary Information

## Figures and Tables

**Table 1:** Calculated exposure values for the experiments.

	Max PP	Shots PP within 3 dB max	Shots with PP > 200	Max SEL	Shots SEL within 3 dB max	Shots with SEL > 180	Max SEL_cum_	Median SEL_cum_
45 in^3^	209	2	13	186	2	3	192	191
150 in^3^ low pressure	210	1	11	189	1	7	193	192
150 in^3^ high pressure	212	3	38	190	3	25	199	197

Given are: maximum peak-peak (pp, dB re 1 μPa); number of signals within 3 dB of maximum pp; number of signals > 200 dB re 1 μPa pp; maximum sound exposure level (SEL, dB re 1 μPa^2^.s); SEL within 3 dB of maximum SEL; number of signals > 180 dB re 1 μPa^2^.s SEL; maximum cumulative SEL (SEL_cum_, dB re 1 μPa^2^.s); median SEL_cum_. Counts of shots exceeding a threshold were for the pot with the highest value.
