# Supplementary material for: Seismic air gun exposure during early-stage embryonic development does not negatively affect spiny lobster Jasus edwardsii larvae (Decapoda:Palinuridae)
Source: Sci Rep. 2016 Mar 7;6:22723. doi: 10.1038/srep22723 (PMC4779986; doi:10.1038/srep22723)
Supplement: Supplementary Information [file srep22723-s1.docx]

**Seismic air gun exposure during early-stage embryonic development does not negatively affect spiny lobster *Jasus edwardsii* larvae (Decapoda:Palinuridae)**

Ryan D. Day^1,*^, Robert D. McCauley^2^, Quinn P. Fitzgibbon^1^, Jayson M. Semmens^1^

^1^ Fisheries and Aquaculture Centre, Institute for Marine and Antarctic Studies, University of Tasmania, Hobart, Tasmania, Australia

^2^ Centre for Marine Science and Technology, Curtin University, Perth, Western Australia, Australia

*Corresponding author: ryan.day@utas.edu.au

**Supplementary Information**

*Air gun methods*

The three experiments had sources of: a) 45 in^3^ air gun operated at 2000 psi (1); b) 150 in^3^air gun operated at 1300 psi (2); c) 150 in^3^air gun operated at 2000 psi (3); and d) the air gun deployed as previously and run across the site but not operated (control). The measured signal levels are shown in Figure 1 of the main text. While system gains were set up correctly, problems with diode protection used on the sea noise logger pre-amplifier input meant that only for the trial with the 150 in^3^air gun at 2000 psi were measured levels accurate at ranges < 100 m. Each experiment involved multiple lobster pots (20, 20 and 10 for the 45 in^3^, 150 in^3^ low pressure and 150 in^3^ high pressure experiments respectively) spaced along a line extending100-250 m north-south (see Supplementary Figure 1) thus measurements from the sea noise loggers were used to build relationships of received level (peak to peak and sound exposure level or SEL) for that source with range and to use this relationship to estimate all fired air gun signal levels at each lobster pot. The estimated received levels were used in a statistical fashion for all pots to define the exposure regime for the respective experiment, based on levels received at each pot.

A Curtin air-gun source model was used to estimate source levels (at one-m) of the air gun configurations used, which for the 45 in^3^ @ 2000 psi, 150 in^3^ @ 1300 psi and 150 in^3^ @ 2000 psi respectively were: peak-peak 223, 224 and 227 dB re 1µPa; and SEL 200, 203, 205 dB re 1µPa^2^.s . The near field hydrophone was not accurate for measuring air gun source level as it was too close to the source. A curve was fitted to the measured levels (peak-peak and sound exposure level independently) of the 150 in^3^ @ 2000 psi data, using: a) the mean value in logarithmic range bins; and b) of the form

$$RL=a\log_{10}\left(R\right)+b*R+SL$$

where *RL* is received level, *R* is range, *SL* is the (fixed) source level and *a* & *b* are values derived from the data. The measured curve a) above described the anomalies in the transmission for the site (due to environmental factors) but was less accurate at ranges < 20 m where the data was scarce. For peak-peak and SEL the two curves agreed over the range 10-20 m so a hybrid curve was used, with ranges < 20 m using the curve b) and ranges > 20 m using curve a). The curve was adjusted for the difference in source level according to the air gun source model for the 45 in^3^ and 150 in^3^ low-pressure sources to give six sets of curves to predict peak-peak and SEL for the three sources. These curves are shown on Figure 1 of the main text.

The range of source to receiver was then used to estimate received level (peak-peak and SEL) at each pot during each experiment from which the statistics given in Table 1 in the main text were derived. The cumulative SEL (CSEL or sum of sound exposure values in linear units of all air gun shots received, expressed in dB values) were calculated for each pot, with the median and maximum CSEL values derived using data for the different pots. Maximum exposures received depended on pot proximity to the air gun, which was random amongst experiments, but the cumulative sound loading, or total dose of sound received, was highest for the 150 in^3^@ 2000 psi experiment as given by the number of signals which exceeded set thresholds and the median CSEL amongst pots (Table 1 in the main text) and the distribution of peak-to-peak and SEL levels are given in Supplementary Figure 3.

A single air gun was used in experiments, a Sercel G Gun II with a 45 in^3^chamber used in 2013 and a 150 in^3^chamber used in 2014. In 2013 the air gun was towed at 5.1 m depth 17 m astern the 5.2 m vessel *RV* *Morana* (aluminium work boat, twin 250 HP 4 stroke motors). In 2014 the air gun was towed at 5.1 m depth, 17-18 m astern the vessel *FV ShellyTon*, an 11 m fishing vessel (beam 3.95 m, 10 tonnes gross, 400 HP single screw). Two GPS units logging every 1 s were mounted side by side, inboard of the respective vessel, with the aerial and tow offsets used to calculate air gun location. The air gun was operated every 11.6 s at a mean speed of 1.85 ms^-1^ or 3.60 knots. A near field hydrophone (HTIU-90) was located 0.5 m off the gun ports and all near field air gun signals logged to a Sound Devices SD722 or SD744 digital recorder, using a -20 dB pre-amplifier and -6 dB gain on the recorder and 24 bit, 48 kHz sampling. The air gun was operated from a bank of G size high pressure air bottles (350 Bar or 35 MPa rated, two bottles in 2013, four bottles in 2014), In 2013 a single Bauer 0.19 m^3^min^-1^ compressor was used, while in 2014 twin compressors were operated in parallel, a Bauer-70, 0.66 Lmin^-1^ and a Munchen 0.57 Lmin^-1^. The system and gas bottles were pumped to 300 Bar with all safety relief valves set at 350 Bar. Approximately 120 or 110 shots at full pressure (2000 psi, 13.8 MPa) were available with full gas bottles and the compressor/s running using the 45 in^3^and 150 in^3^setup (2013 and 2014 respectively). The time taken to fill bottles was highly dependent on ambient temperature. An air gun firing control system which triggered a log file via TTL output on a firing pulse was used. The minimum vessel crew was four, skipper, marine mammal observer and two air gun operators. The air gun was deployed and recovered charged via a lifting davit.

To monitor the air gun signal exposure received by target animals and the normal ambient noise regime at the site, sea noise loggers were set on the seabed over the full experimental duration. All sea noise loggers recorded pressure while two recorded ground borne vibration via geophones (not analysed here). The sea noise loggers were located next to lobster pots at each end of the pot lines during experiments. The sea noise loggers were Curtin University designed, CMST-DSTO sea noise recorders (www.cmst.curtin.edu.au/products). All noise loggers had pressure sensors fitted using High Tek HTI U90 or Massa TR1025C hydrophones. Two noise loggers were modified to include 3-axis geophone sensors to measure ground borne vibration. Two geophones were aligned at opposing 45^o^ angles from the horizontal and one was aligned vertically. All sea noise logger housings were placed on the seabed by divers. The housings were stainless steel, 6 mm wall thickness and had plastic cross bars with weights at each end to stop the housing rolling. The weight of the housing, cross bar and batteries (~ 50 kg underwater) ensured the housing was firmly coupled to the seabed. The hydrophones were external to the housing and sat freely on the seabed. The hydrophone cable was weighted to prevent it moving. All sea noise recorders were calibrated for the pressure response by inputting white noise of known level (traceable standard) into the logger with the white noise source and hydrophone in series. Analysis of the logged signal gave the system gain with frequency, accounting correctly for the impedance match of the hydrophone, pre-amplifier and system electronics. This system gain curve was used with the known hydrophone sensitivity to convert the logged volts to Pa with the system response calibrated over 1 Hz to the anti-aliasing filter frequency. The on-board noise logger clocks were set to GPS, UTC transmitted time before deployments using hardware and software and the drift read after recovery to give absolute timing accuracies of < 0.1 s.

In 2013 and the first trial of 2014, 20 lobster pots were set over approximately 250 m north-south on the reef substrate at the eastern end of the channel separating Betsy Island and Blackjack Reef. Dan-buoys marked the end of the lines of pots with each pot having a small surface buoy. Noise loggers were located at the end of the lobster pot lines and in the line centre (2013). For the second trial in 2014 only 10 pots were deployed. Locations of the control and exposed runs for all lines and the pot locations in 2013 and 2014-1 are shown on Supplementary Figure 2 with details of the experiments listed in Supplementary Table 1.

*Analysis and units*

All times given here are Australian Eastern Standard Time (UTC + 10 hours). All air gun and spatial analysis has been carried out in the Matlab environment using purpose built software. Air gun signals were analysed by: 1) extracting the signals from the sea noise logger files; 2) converting volts to sound pressure (Pa) using the system calibration curve and hydrophone sensitivity in the time domain; 3) characterising the air gun signal for 16 signal parameters as defined in McCauley et al (2003); and 4) aligning the shot received time with the source navigation data to give the source-receiver, slant-range (direct path source to receiver, not horizontal range).

Supplementary Table 1: Details of air gun experiments, listing: nominal name; date; time of control start and end with elapsed time; time of first and last air gun shots with elapsed time; number of air gun shots; and the source used.

| **expt.** | **Date** | **start** | **end**  **(minutes)** | **shots** | **source** | **Speed (ms^-1^, kn)** |
| --- | --- | --- | --- | --- | --- | --- |
| Lobster-1  control | 03-Jul-2013 | 09:56:21 | 10:14:10 (17.8) |  | none | 1.69, 3.28 |
| Lobster-1  exposed | 03-Jul-2013 | 11:36:05 | 12:00:35 (24.5) | 134 | 45 in^3^  2000 psi | 1.71, 3.32 |
| Lobster-2  control | 21-Jul-2014 | 11:22:50 | 11:52:50 (30) |  | none | 2.01, 3.91 |
| Lobster-2  exposed | 21-Jul-2014 | 13:35:37 | 13:54:50 * (19.2) | 112 | 150 in^3^  1300 psi | 1.95, 3.80 |
| Lobster-3  control | 28-Jul-2014 | 11:17:51 | 11:45:44 (27.9) |  | none | 1.92, 3.74 |
| Lobster-3  exposed | 28-Jul-2014 | 12:37:23 | 13:00:42 (23.3) | 110 | 150 in^3^  2000 psi | 1.84, 3.57 |


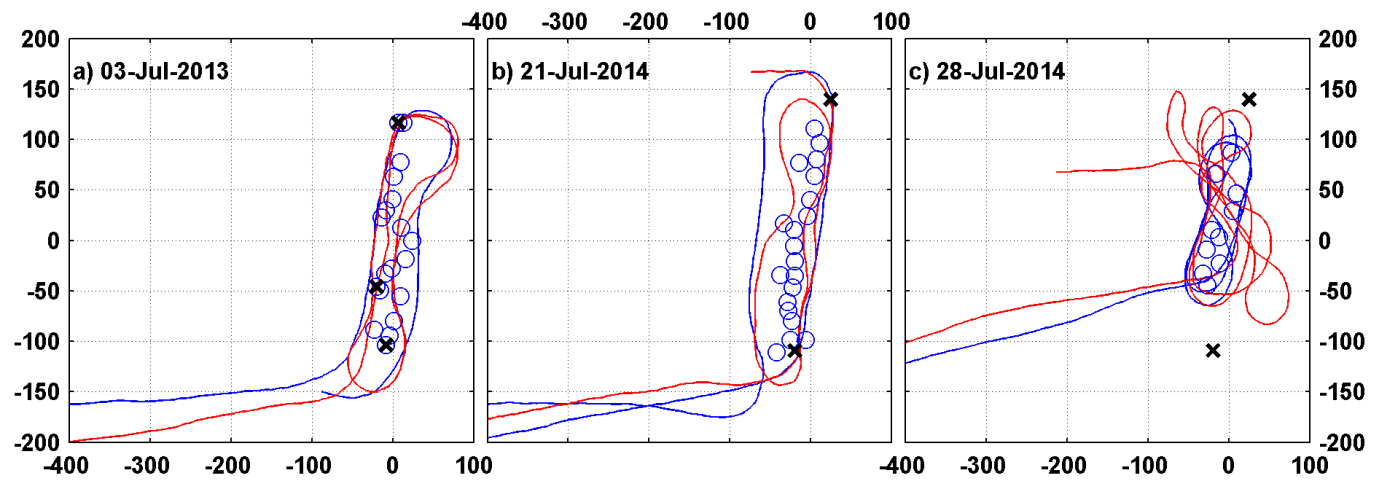


Supplementary Figure 1: Control (blue) and air gun runs (red) during the three lobster experiments 2013-2014. The circles are pot locations and the crosses sea noise logger locations.


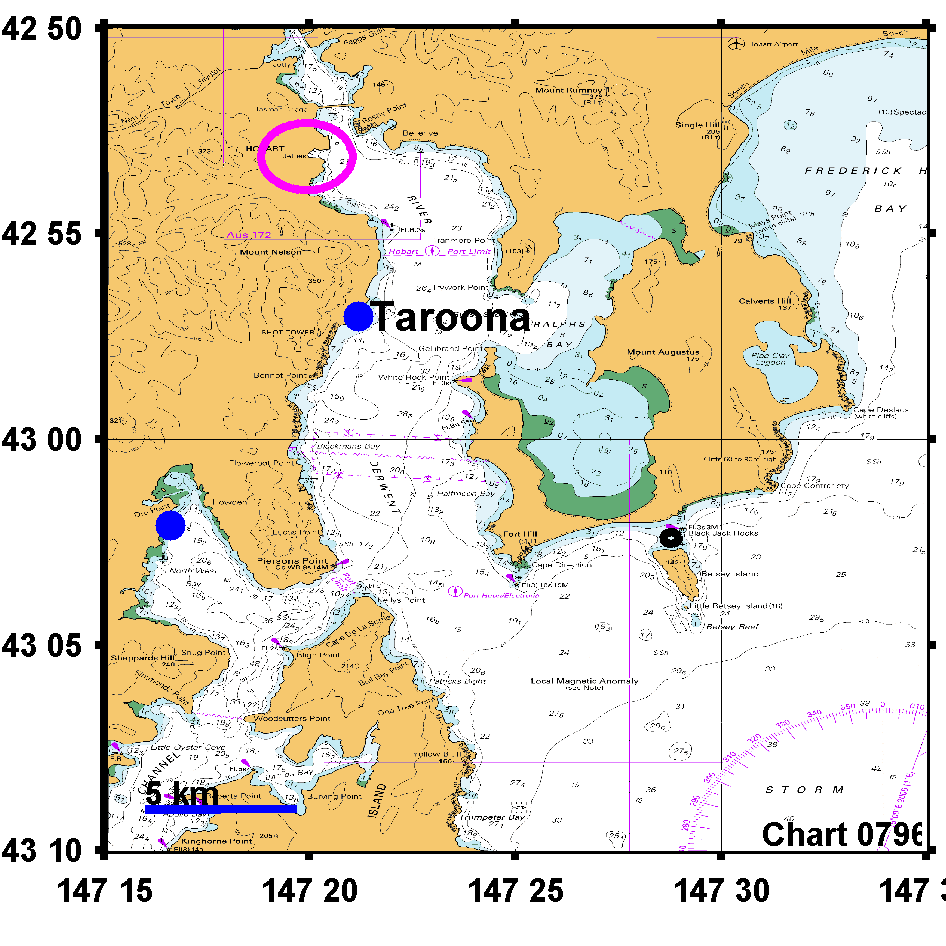

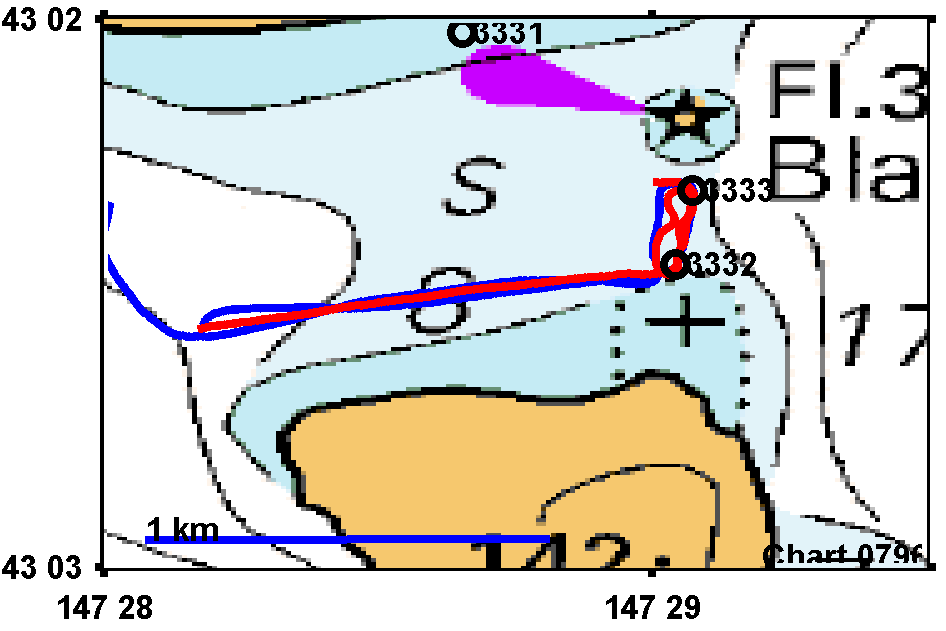


Supplementary Figure 2: (left) Larger scale chart of general study area showing: Hobart area (bounded by magenta circle); Taroona aquaculture facility location; vessel mobilisation port (blue dot on left) and lobster study site (black circle north of Betsey Island). (right) Larger scale chart of lobster study site showing example control (blue curve) and air gun path (red curve) made on 21-Jul-2014 and locations of sea noise loggers (sets 3331, 3332 and 3333). The lobster pots were laid out between noise loggers 3332 and 3333. Maps were generated in MatLab from Australian Hydrographic Service charts under Seafarer GeoTIFF license No 2618SG.


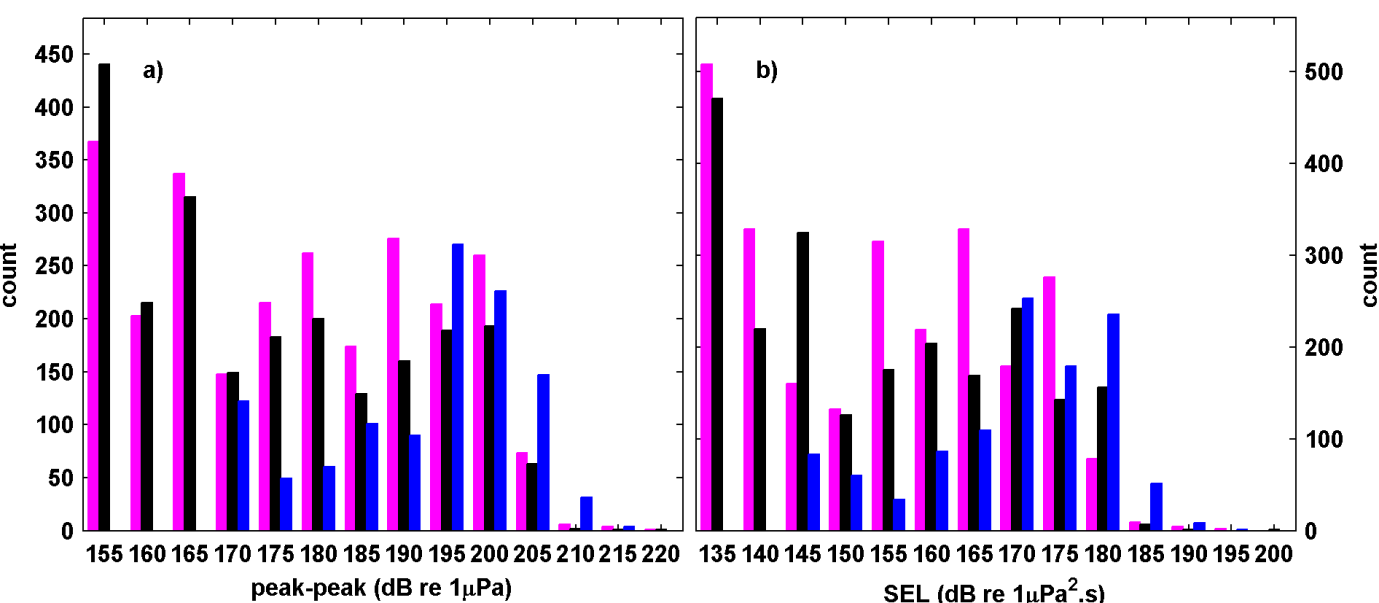


Supplementary Figure 3: Distribution of estimated levels at all pots for the three experiments (45 in^3^@ 2000 psi magenta, 150 in^3^@ 1300 psi black and 150 in^3^2000 psi blue), with a) peak to peak and b) SEL.
